# KIT-13, a novel plasmalogen derivative, attenuates neuroinflammation and amplifies cognition

**DOI:** 10.3389/fcell.2024.1443536

**Published:** 2024-09-02

**Authors:** Md Shamim Hossain, Shiro Mawatari, Masanori Honsho, Tatsuo Okauchi, Takehiko Fujino

**Affiliations:** ^1^ Division of Lipid Cell Biology, Institute of Rheological Functions of Food, Fukuoka, Japan; ^2^ Department of Neuroinflammation and Brain Fatigue Science, Graduate School of Medical Sciences, Kyushu University, Fukuoka, Japan; ^3^ Department of Applied Chemistry, Kyushu Institute of Technology, Fukuoka, Japan

**Keywords:** plasmalogen derivative, neuroinflammation, cognitive enhancement, neurogenesis, Alzheimer’s disease

## Abstract

Plasmalogens (Pls) are specialized phospholipids integral to brain health, whose decline due to aging and stress contributes to cognitive impairment and neuroinflammation. This study explores the potential of a novel Pls derivative, KIT-13 (1-O-octadecyl-2-arachidonoyl-sn-glycerol-3-phosphoethanolamine), in mitigating neuroinflammation and enhancing cognition. When administered to mice, KIT-13 exhibited potent memory enhancement attributed to upregulated brain-derived neurotrophic factor (BDNF), a key player in cognitive processes. *In vitro* experiments with neuronal cells revealed KIT-13’s ability to induce robust cellular signaling, surpassing natural plasmalogens. KIT-13 also promoted neurogenesis and inhibited apoptosis of neuronal-like cells, highlighting its potential in fostering neuronal growth and plasticity. Additionally, KIT-13 treatments reduced pro-inflammatory cytokine expression and attenuated glial activation in the brain. KIT-13’s superior efficacy over natural Pls positions it as a promising therapeutic candidate for neurodegenerative conditions such as Alzheimer’s disease, characterized by cognitive decline and neuroinflammation. This study presents KIT-13 as an innovative approach for addressing cognitive impairment and neuroinflammatory pathologies.

## Highlights

⁃ Innovative Compound: KIT-13, a novel alkyl-type plasmalogen derivative, demonstrates superior efficacy in cellular signaling and neurogenesis compared to natural plasmalogens.
⁃ Neuroprotective Effects: KIT-13 significantly reduces apoptosis in neuron-like cells, showcasing its potential as a robust neuroprotective agent.
⁃ Cognitive Enhancement: Administration of KIT-13 in adult c57BL6J (B6) male mice led to substantial improvements in learning and memory, indicating its promise for treating cognitive impairments.
⁃ Neuroinflammation Reduction: KIT-13 effectively attenuates neuroinflammation, reducing pro-inflammatory cytokine expression and glial activation in the brain.
⁃ BDNF Stimulation: KIT-13 treatment results in heightened expression of brain-derived neurotrophic factor (BDNF), which is crucial for cognitive processes and neuronal health.
⁃ Amyloid Beta Reduction: In neuroinflammation models, KIT-13 shows remarkable efficacy in reducing the accumulation of amyloid beta, a hallmark of Alzheimer’s disease.
⁃ Potential Therapeutic: KIT-13’s superior performance over natural plasmalogens positions it as a promising therapeutic candidate for neurodegenerative conditions like Alzheimer’s disease.

## Introduction

Plasmalogens, a subclass of glycerophospholipids, are characterized by the presence of a distinctive vinyl ether bond at the sn-1 position of the glycerol backbone. (Farooqui and Horrocks, 2001; Grimm et al., 2011; Braverman and Moser, 2012; Sejimo et al., 2018; Hossain et al., 2020; Saito et al., 2023). These phospholipids play critical roles in membrane structure and function, and are involved in various biological processes, including cell signaling, antioxidative defense, and membrane fusion. Despite their significance, plasmalogens are particularly susceptible to degradation in the gastrointestinal tract (GIT) due to their sensitive vinyl ether bond. This enzymatic breakdown limits their direct application and efficacy when considering therapeutic delivery strategies. Alkyl-type glycerophospholipids offer a more robust alternative to their vinyl ether-containing counterparts (Honsho and Fujiki, 2023). Their ether bond at the sn-1 position provides greater resistance to enzymatic degradation in the GIT, enhancing their stability and ensuring that a higher proportion reaches systemic circulation intact. Once absorbed, these alkyl-type glycerophospholipids can undergo enzymatic conversion to alkenyl-type plasmalogens, effectively restoring plasmalogen levels in tissues and eliciting the desired biological effects (Dorninger et al., 2022; Honsho and Fujiki, 2023). Advancing our knowledge in this field could pave the way for novel therapeutic strategies aimed at treating diseases associated with plasmalogen deficiency or dysfunction.

The pathological studies showed that ethanolamine-type plasmalogens (PlsEtn) are reduced in brain of patients with Alzheimer’s disease (AD), suggesting a possibility that reduction of these lipids could be associated with loss of cognitive function (Senanayake and Goodenowe, 2019; Su et al., 2019). Although the direct impact of reduced brain Pls on the progression of memory dysfunction in AD patients is not fully understood, mouse studies have demonstrated a significant reduction in learning and memory processes when hippocampal Pls levels are decreased (Hossain et al., 2022b). This reduction of Pls in hippocampus tissues resulted in increased neuroinflammation and decreased BDNF signaling. Pls levels are not only reduced by the aging process but also by neuroinflammation and stress (Hossain et al., 2017). The mechanism underlying this Pls reduction is linked to the transcriptional downregulation of the Pls synthesizing gene, GNPAT (Hossain et al., 2017). Inducing neuroinflammation through LPS injection in mice has been shown to impair memory (Hossain et al., 2018; Zhao et al., 2019), suggesting that neuroinflammation could be directly related to memory loss and Pls reduction. Neuroinflammation is often considered as one of the main events for the cognitive decline in patients with AD (Kumar, 2018; Passamonti et al., 2019; Lecca et al., 2022).

Oral ingestion of natural Pls (Pls extracted from scallop, sPls) was shown to enhance memory and inhibit neuroinflammation in mice (Ifuku et al., 2012; Hossain et al., 2018; Hossain et al., 2020; 2022b; 2023). Clinical studies have also reported improved cognitive function in AD patients following oral ingestion of sPls (Fujino et al., 2017; Fujino et al., 2020), indicating that oral administration of Pls and their derivatives could be a potent therapeutic strategy for improving cognition in AD patients. However, the role of alkyl-type plasmalogen derivatives in memory formation remains largely elusive. Given the importance of memory improvements among neurodegenerative diseases, including AD, plasmalogen derivatives could have a significant therapeutic impact due to their stable nature.

In mouse experiments, Pls improved spatial memory in association with increased neurogenesis and enhanced brain derived neurotrophic factor (BDNF) signaling pathway (Hossain et al., 2022b). Previous findings also indicate that Pls treatments inhibit apoptosis and enhance ERK signaling in neuronal cells (Hossain et al., 2013; Hossain et al., 2020). Pls treatments have been shown to rescue learning and memory deficits in LPS-induced neuroinflammation model mice by attenuating glial activation (Hossain et al., 2018). These *in vivo* studies are further supported by findings that direct application of sPls inhibits pro-inflammatory cytokine induction in microglial cells derived from mice. These compelling findings underscore the potential of Pls and their derivates as a transformative therapeutic strategy for neurodegenerative diseases. Developing synthetic Pls derivatives that exhibit similar functions to natural Pls is crucial for future therapeutic strategies targeting Pls deficiencies. Several Pls derivatives have shown promising beneficial effects, particularly in Parkinson’s disease model mice (Fallatah et al., 2020). In our screening process, we analyzed various alkyl-type Pls derivatives and identified KIT-13 (an alkyl Pls with arachidonic acid at the sn-1 position) as the most effective, showing even better effects than sPls.

## Materials and methods

### Plasmalogens and the derivative KIT-13

Scallop-derived plasmalogens (sPls), which were previously described (Fujino et al., 2020; Hossain et al., 2022b), were used as the control group to examine the effects of the Pls derivative KIT-13. KIT-13 is an alkyl-acyl ethanolamine plasmalogen precursor with stearic alcohol at the sn-1 position and arachidonic acid at the sn-2 position. The full chemical name is 2-[(((hydroxyR)-2-((5Z,8Z,11Z,14Z)-icosa-5,8,11,14-tetraenoyl)oxy)-3-((octadecyloxy)propoxy) phosphoryl)oxy] ethan-1-aminium and the generic name is 1-O-octadecyl-2-arachidonoyl-sn-glycerol-3-phosphoethanolamine. The KIT-13 was synthesized and purified at Kyushu Institute of Technology, Japan. Its structure was confirmed through Nuclear Magnetic Resonance (NMR) and Mass Spectrometry assays. The synthesis paper of KIT-13 is currently under submission, and details of the synthesis process will be provided upon proper request.

### Cell culture

The Neuro2A, SH-SY5Y, and the microglial cell line (MG6) were purchased from RIKEN Cell Bank. The cells were cultured in DMEM medium supplemented with bovine calf serum (10%), as mentioned in previous articles (Hossain et al., 2022b).

### ELISA assays

The ELISA assays were performed according to the standard protocols provided by the ELISA kit providers: p-ERK (FastScan phospho-p44/42 MAPK, 42,173, Cell Signaling), p-Akt (FastScan phosphor-Akt Ser-473, 80,895, Cell Signaling), IL-1beta (mouse IL-1 beta DuoSet ELISA, DY401, R&D Systems), TNF-alpha (mouse TNF-alpha DuoSet ELISA, DY410, R&D Systems), and MCP-1 (mouse MCP-1 DuoSet ELISA, DY479, R&D Systems). To assess p-ERK and p-Akt (Ser-473) expression in Neuro2A cells, we treated the cells with KIT-13 and sPls at a concentration of 5 μg/mL for 10 min. Subsequently, proteins were extracted from whole cell lysates using 1X cell extraction buffer provided with the Cell Signaling ELISA kit. For the ELISA assays, we prepared the capture antibody, HRP-linked antibody, TMB substrate, and STOP solutions according to the protocol provided in the ELISA kits. A total of 10 µg of protein from each sample was subjected to the ELISA assays. Spectrophotometric measurements were performed within 30 min after adding the STOP solution at an absorbance of 450 nm. The expression of cytokines (IL-1beta, TNF-alpha, and MCP-1) was normalized with the equal number of cells and the same quantity of brain tissues. When used brain tissues, we used sarcosine lysis buffer (0.1% sarcosine, 50 mM Tris-Hcl pH7.5, and 150 mM NaCl) to dissolve the hippocampus tissues with sonication on cold water and equal amount of the dissolved protein (10 µg) was subjected to the ELISA assays. The absorbance reading was measured by DTX 880 Multimode Detector.

### Apoptosis assays

The apoptosis assays utilizing the TUNEL method were executed following the provided guidelines from the *in-situ* Cell Death Detection Kit, TMR red (Roche), as mentioned in the published paper (Hossain et al., 2013). After the experimental treatments, cells underwent fixation in a 4% paraformaldehyde solution and were washed with PBS. To enable permeabilization, a solution containing 0.1% Triton X-100 in freshly prepared 0.1% sodium citrate was employed. The subsequent step involved treating the cells with the TUNEL mixture, with an incubation period of 1 h at 37°C. Any remaining TUNEL reagents were meticulously removed through thorough washing with cold PBS. Finally, a mounting solution containing DAPI (1 μg/mL) was used. The cells were then mounted and subjected to analysis using fluorescence microscopy (Axioskop 2, Zeiss) to observe and quantify the outcomes of the TUNEL-based apoptosis assays.

### ELISA assays for detecting cleaved Caspase-3

The detection of cleaved caspase-3 was carried out using the DuoSet IC ELISA kit (Human/Mouse Cleaved Caspase-3 Asp175, DYC835, R&D Systems), following the manufacturer’s protocol. Neuro2A cells were treated with sPls and KIT-13 at a concentration of 5 μg/mL for 48 h in serum-free DMEM medium. For the control group, cells were cultured in DMEM medium containing 10% FBS. Post-treatment, cells were washed and proteins were extracted using the lysis buffer provided in the ELISA kit. A total of 20 µg of protein extract was used for each ELISA reaction to measure cleaved caspase-3 levels. Prior to the ELISA assays, protein samples were diluted with the diluent included in the kit. The absorbance was recorded at 450 nm using the DTX 880 Multimode Detector after the addition of the STOP solution.

### Animal studies and memory assays

All animals were maintained according to the established protocols outlined by the ethical standards set forth by MONBUKAGUSHO in Japan. Prior to procuring the mice, the ethical committee of Rheology granted formal approval for the mouse experiments. Specifically, male B6 (c57BL6J) mice, aged 12 weeks, were selected for the study. To assess the effects, the mice were orally administered KIT-13 at a dose of 10 mg/50 kg/day for a duration of 30 days. During this period of oral administration, mice concurrently underwent intraperitoneal injections of the inflammatory agent LPS (25 mg/kg/day) for a duration of 7 days. The water maze tests and probe tests were conducted in accordance with established methods as described before (Hossain et al., 2018; Hossain et al., 2022b). As a reference point, a control group received neither LPS nor KIT-13. Each experimental group comprised 10 mice (n = 10) for comprehensive analysis. To assess the memory of mice following the training (learning) periods, mice were subjected to probe tests on the day after finishing the training trials. In the probe tests, we recorded the time it took the mice to reach the platform. For the neurogenesis assays, the mice (9 weeks old B6) were starved of water for 12 h followed by oral ingestion of KIT-13 (10 mg/50 Kg) for 24 h.

### Immunofluorescence studies

The cells were cultured on glass coverslips and underwent immunocytochemistry (ICC) using the following protocol. First, cells were fixed with 4% PFA for 20 min at room temperature, followed by three PBS washes (5 min each). Subsequently, cells were incubated with a blocking buffer containing the cell-permeabilizing detergent Triton-X100 for 1 h. This blocking buffer consisted of 0.2% Triton X-100% and 5% BSA dissolved in PBS. Following three PBS washes, cells were then exposed to primary antibodies BDNF (1:2000, Abcam), and DCX (1:2000, Santa Cruz) dissolved in the blocking buffer. This incubation occurred at 4°C for 24 h. After a PBS wash, cells underwent treatment with Alexa Fluor 488/568-conjugated secondary antibodies (1:1,000, Life Technologies) dissolved in the blocking buffer for 2 h at room temperature. After the incubation with secondary antibodies, cells were washed and mounted with DAPI-containing mounting medium. The immunohistochemistry (IHC) studies were followed by the previously described methods (Hossain et al., 2017). The following antibodies were used IHC studies: Iba1 (Wako), Cy3 conjugated GFAP (Millipore), amyloid beta (Aβ1-16, Clone 6E10, BioLegend), and DCX (Santa Cruz, E-6, sc-271390). The images were captured using a fluorescence microscopy (Axioskop 2, Zeiss).

### Western blotting studies

The monoclonal antibodies against β-Actin and doublecortin (DCX) were purchased from MBL (MBL M177-3) and Santa-Cruz, respectively. Proteins were extracted from hippocampal tissues using a 0.5% sarcosine lysis buffer. Equal quantities of protein (30 µg) were separated on a 10% SDS-PAGE gel and then transferred to a ClearTrans Nitrocellulose Membrane (0.2 µm, 030-25643, Wako). The membrane was treated with primary antibodies at the following dilutions: β-Actin (1:4,000) and DCX (1:1,000). After washing, the membranes were incubated with HRP-coupled goat anti-mouse IgG secondary antibodies (Cell Signaling) at room temperature for 2 h. Protein signals were visualized using the Western Lighting Plus-ECL system (PerkinElmer, United States) and LumiCube (Liponics, Japan).

## Results

### KIT13 enhanced cellular signaling, reduced cytokine expression and enhanced BDNF expression

**FIGURE 1:**
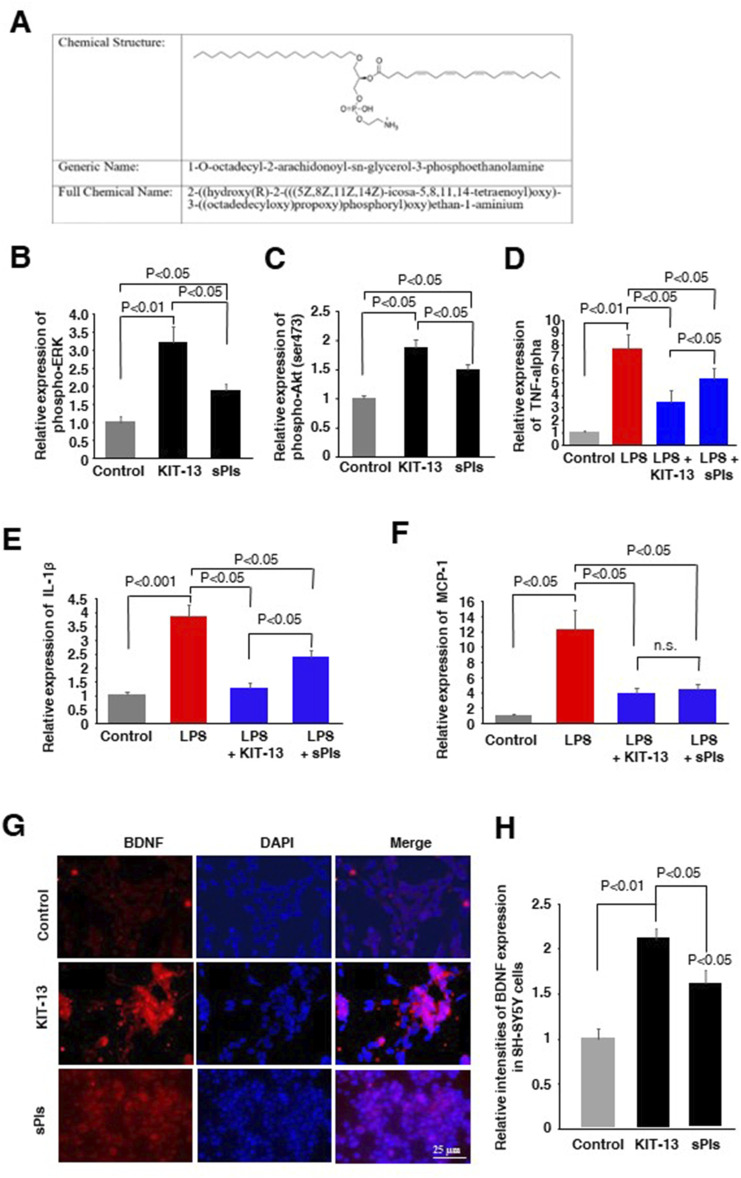
KIT-13 enhances cellular signaling, reduces neuroinflammation, and induces BDNF expression. **(A)** Chemical structure of KIT-13 (1-O-octadecyl-2-arachidonoyl-sn-glycerol-3-phosphoethanolamine). **(B,C)** ELISA assays of phosphor-ERK and phosphor-Akt on Neuro2A cell extracts treated with KIT-13 and sPls (5 μg/mL) for 24 h. **(D–F)** Relative expression of TNF-alpha **(D)**, IL-1β(E), and MCP-1 (F) in the culture medium of MG6 cells pretreated with KIT-13 (5 µg/mL) and sPls (5 µg/mL) for 12 h, followed by LPS (1 µg/ml) treatment for an additional 6 h. **(G)** Immunocytochemistry (ICC) assays showing BDNF expression in SH-SY5Y cells treated with KIT-13 and sPls (5 µg/mL) for 36 h. Scale bar = 25 µm. **(H)** Quantification of BDNF expression from ICC assays in panel F. All statistical analyses were performed using ANOVA followed by post hoc Bonferroni tests. Panels B to E: n = 5 experiments per group. Panels **(G, H)**: n = 3 experiments per group, with 10 fluorescence images analyzed per experiment using ZEN 3.3 software. Values represent the mean ± SEM.

In the present study, we synthesized a new plasmalogen derivative, 1-O-octadecyl-2-arachidonoyl-sn-glycerol-3-phosphoethanolamine, named KIT-13 (Figure 1A), to screen its biological effects in comparison to naturally extracted scallop plasmalogen (sPls). It is well established that Plasmalogens derived from scallops (sPls) enhance cellular signaling pathways, including ERK and AKT (Hossain et al., 2013; Hossain et al., 2016). To investigate whether the plasmalogen derivative, KIT-13, exhibits similar effects on cellular signaling as natural plasmalogens, we conducted experiments on Neuro2A cells, consistent with previous studies (Hossain et al., 2013; Hossain et al., 2016). Treatments with KIT-13 resulted in a greater enhancement of ERK and Akt protein phosphorylation than treatments with natural plasmalogens (sPls), as confirmed by ELISA assays (Figures 1B, C). In addition to the enhancement of cellular signaling, plasmalogens were reported to show anti-inflammatory effects in microglial cells (Ifuku et al., 2012; Hossain et al., 2017; Ali et al., 2019; Ali et al., 2022). To assess the anti-inflammatory properties of direct KIT-13 application and its resemblance to sPls, we examined the expression of TNF-alpha, IL-1β and MCP-1 (pro-inflammatory cytokines) in mouse-derived microglial cells (MG6) following LPS treatments, as described previously (Youssef et al., 2019). Our findings demonstrated that KIT-13 treatments led to a greater reduction in cytokine expression than sPls (Figures 1D–F). Previously, we discovered that sPls augmented BDNF expression, which was linked to memory improvement (Hossain et al., 2022b). To investigate whether KIT-13 could increase BDNF expression, we conducted *in vitro* studies using SH-SY5Y cells. This cell line is widely utilized to study the regulation of BDNF expression (Gonulalan et al., 2018; de Medeiros et al., 2019; Zhang et al., 2021). Our study revealed that prolonged exposure to KIT-13 induced greater BDNF expression than sPls treatments (Figures 1G, H). Although the mechanism by which KIT-13 induces BDNF expression is not yet fully understood, it is likely that the activation of ERK and Akt pathways could play a significant role in increasing the expression of this memory-associated neuropeptide in cells. These findings strongly suggest that KIT-13 exhibits comparable, if not superior, effects compared to sPls treatments.

### KIT-13 inhibited neuronal apoptosis

**FIGURE 2:**
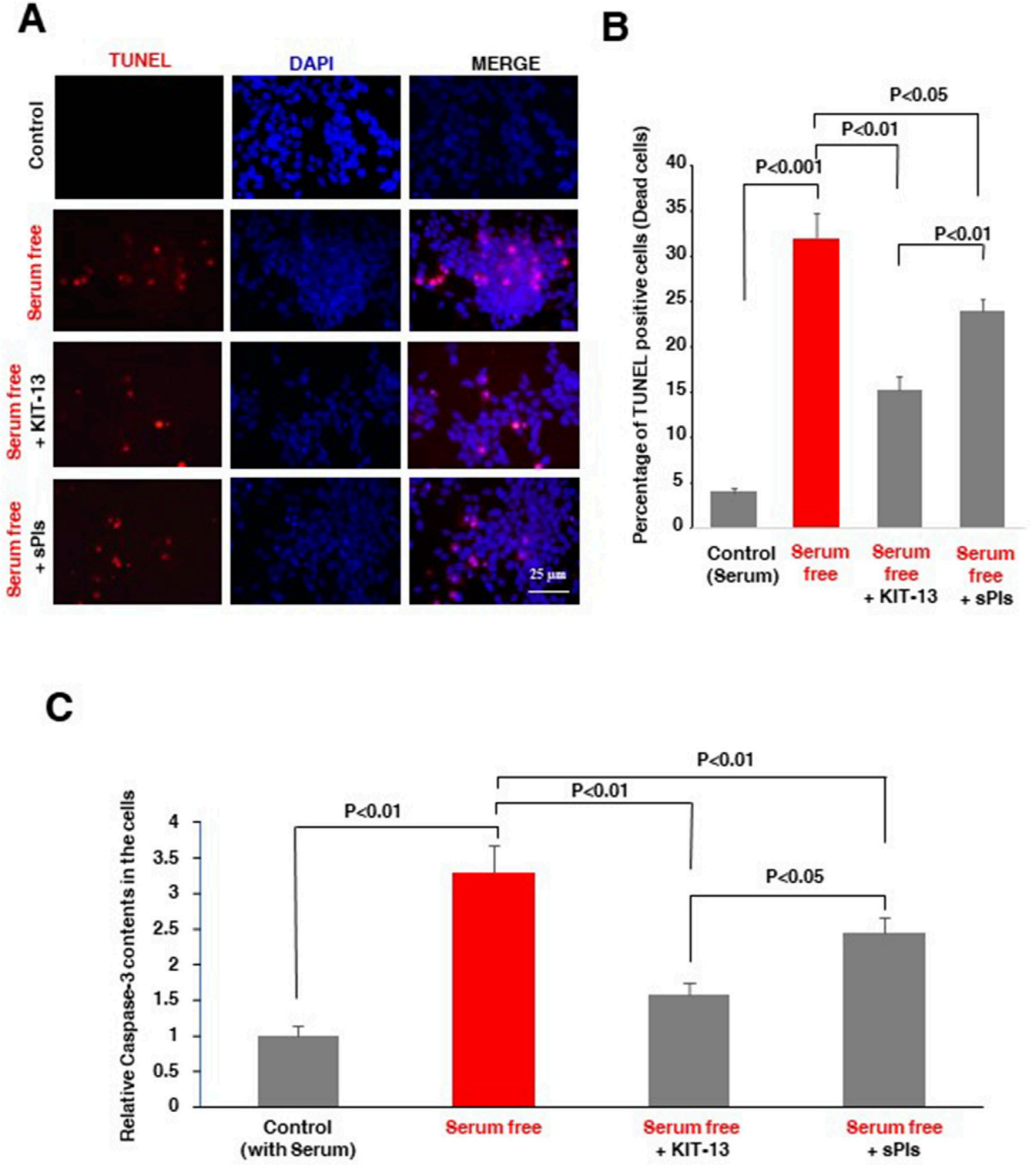
KIT-13 prevents apoptosis in Neuro2A cells under serum starvation **(A)** Neuro2A cells were treated with KIT-13 and sPls (5 μg/mL) for 36 h in serum-free medium. Apoptotic cells were detected using the *in-situ* cell death detection kit, and nuclei were stained with DAPI. Scale bar = 25 µm. **(B)** Quantification of apoptotic cells from panel A, showing the extent of apoptosis prevention by KIT-13. *P*-values were calculated using ANOVA followed by post hoc Bonferroni tests. Each group consisted of three independent experiments (n = 3). Values represent the mean ± SEM. **(C)** ELISA assays showing the relative changes in protein expression of cleaved caspase-3 among the groups described in panel A. The bars indicate the average values of three independent experiments (n = 3) and the error bars represent the standard error of the mean. *P*-values were calculated using ANOVA followed by post hoc Bonferroni tests.

Natural plasmalogens have been demonstrated to inhibit neuronal apoptosis in cells subjected to serum starvation (Hossain et al., 2013). To investigate whether KIT-13 possesses a similar effect, we treated Neuro2A cells with both KIT-13 and sPls in serum free medium for 36 h. Compared to the control group, which was not subjected to serum starvation, KIT-13 treatments significantly reduced apoptosis, as confirmed by TUNEL assays (Figures 2A, B). This reduction was greater when compared to both the control serum-free and sPls treatment groups. To further validate the apoptosis event, ELISA assays were performed to detect the pro-apoptotic marker cleaved caspase-3. Consistent with the TUNEL assays, serum starvation for 36 h significantly increased cleaved caspase-3 protein expression in these cells compared to the control group (no serum starvation). KIT-13 treatment reduced this apoptosis (Figure 2C). KIT-13 demonstrated a better effect than the sPls treatment in serum-free conditions, suggesting that KIT-13 has the potential to inhibit cellular apoptosis during serum starvation. To understand the mechanism by which KIT-13 inhibits apoptosis, we suggest the hypothesis that KIT-13 treatments might reduce the activation of caspase-9, a marker of the mitochondrial-mediated intrinsic apoptosis pathway. In our previous study, we observed that serum starvation increased cleaved caspase-9, which was inhibited by plasmalogen treatments (Hossain et al., 2013). Therefore, it is likely that, similar to sPls, KIT-13 might attenuate mitochondrial stress during serum starvation, which could inhibit neuronal apoptosis.

### KIT-13 enhanced spatial memory and reduced neuroinflammation in mice

**FIGURE 3:**
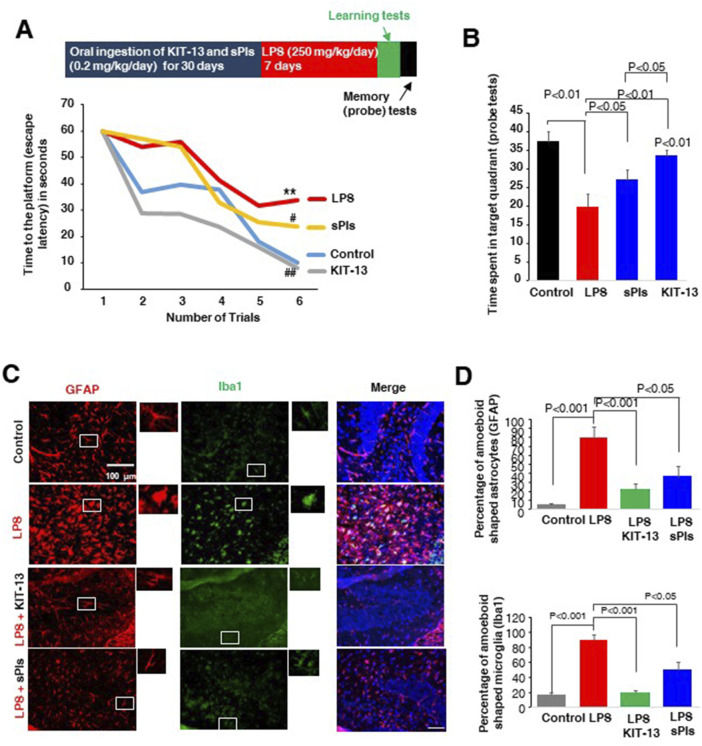
KIT-13 enhances learning and memory in LPS-treated adult mice **(A)** Morris Water Maze Test data showing learning performance over 6 trials (2 trials per day) in mice following treatments. Treatments included 30 days of oral ingestion of sPls and KIT-13 (0.2 mg/kg/day), followed by 7 days of intraperitoneal (i.p.) injection of LPS (250 mg/kg/day). Each group consisted of ten mice (n = 10). * indicates significance (*p* < 0.05) compared to the control group; # (*p* < 0.05) and ## (*p* < 0.01) indicate significance compared to the LPS group. **(B)** Probe tests performed after the learning trials, indicating the time required to reach the platform for each group of mice (n = 10). **(C)** Immunohistochemistry assays showing GFAP and Iba1 positive cells in the hippocampus regions of each group of mice as described in panel A. Scale bar = 100 µm. **(D)** Quantification of amoeboid-shaped glia (GFAP and Iba1 positive cells) in the different groups of mice (n = 5). *P*-values were calculated using ANOVA followed by post hoc Bonferroni tests. Values represent the mean ± SEM.

We previously observed that oral ingestion of natural Plasmalogens rescued LPS-mediated loss of spatial memory in mice (Hossain et al., 2018). Furthermore, oral ingestion of plasmalogens also demonstrated an enhancement of spatial memory in both adult male and aged female mice (Gu et al., 2022; Hossain et al., 2022b). To address whether KIT-13 has the similar effects, we performed the Water maze test in adult male mice orally administered with KIT-13 and sPls. Here we used the dose, 0.2 mg/kg/day, based on the preliminary studies showing the memory improvements effects of sPls in adult male mice (data not shown). After 30 days oral ingestion of KIT-13 and sPls, we treated the mice with the i.p. injection of LPS for 7 days. The LPS injection reduced the learning in the adult mice, which was rescued in KIT-13 pretreatment group more than the sPls group (Figure 3A). The probe tests showed a reduction of memory in the LPS group, which was rescued significantly in sPls and KIT-13 pretreatment groups (*P* < 0.05 and *P* < 0.01 respectively (Figure 3B). Compared to sPls, KIT-13 treatments rescued memory more significantly (*P* < 0.05). To address whether the memory improvement is associated with the reduction of neuroinflammation in murine brain, we performed IHC studies with the brain slices and found that activated microglial cells (Iba1 positive) and astrocytes (GFAP positive) as marked by their amoeboid shape were increased in the LPS group compared to the control group. KIT-13 pretreatments group showed attenuation of the activation of glial cells compared to the sPls group (Figures 3C, D). These results suggest that KIT-13 treatments can rescue LPS-induced activation of glial cells and induction of cytokines in mice brain.

### Oral ingestion of KIT-13 reduced cytokines and accumulation of amyloid beta in hippocampus of neuroinflammation model mice

**FIGURE 4:**
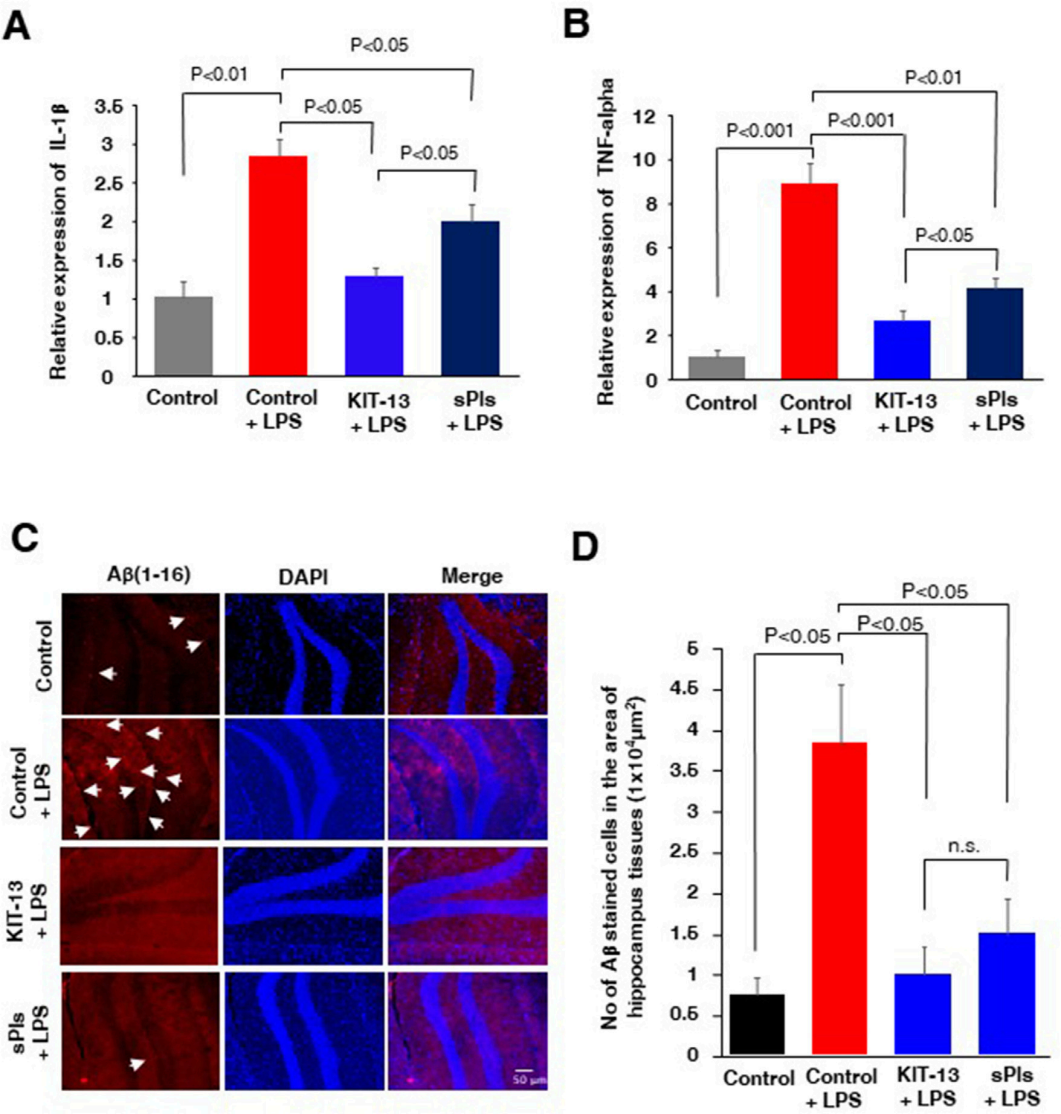
KIT-13 reduced neuroinflammation and amyloid beta expression in adult mice hippocampus **(A, B)** ELISA assays of hippocampus tissues in the mice groups (n = 5) as described in Figure 3, showing the relative expression of the cytokines IL-1β(A) and TNF-alpha **(B)**. Values represent the mean ± SEM. **(C)** Immunohistochemistry assays showing the expression of amyloid beta (Aβ) in the hippocampus areas of mice in each group. Data represent 5 mice per group (n = 5). Scale bar = 50 µm. **(D)** Quantification of Aβ-positive cells in panel C. Ten randomly selected images from each mouse brain were analyzed to count the cells. Mean data were obtained from 5 mice per group, and error bars indicate the SEM. *P*-values were calculated using ANOVA followed by post hoc Bonferroni tests.

It is known that i.p. injection of LPS can increase cytokine expression in brain tissues and lead to an accumulation of amyloid beta (Hossain et al., 2018; Wang et al., 2018; Zhan et al., 2018; Zhao et al., 2019). In our experiments, we assessed cytokine expression in mice hippocampal tissues and observed that i.p. injection of LPS significantly increased the expression of IL-1β and TNF-alpha, which was attenuated in the KIT-13 and sPls pretreatment groups (Figures 4A, B). The KIT-13 treated group showed a greater attenuation in cytokine expression compared to the sPls group, suggesting that KIT-13 is more effective in reducing brain cytokine expression than sPls. Immunohistochemistry (IHC) studies demonstrated that pretreatments with KIT-13 and sPls attenuated the LPS-mediated accumulation of Aβ proteins in the hippocampus tissues (Figures 4C, D). It is known that increased neuroinflammation is linked with AD-like pathologies, including the accumulation of amyloid beta (Hossain et al., 2018; Kinney et al., 2018; Hossain et al., 2020; Wang et al., 2023). Our data suggest that KIT-13 might reduce amyloid beta accumulation in the murine brain, possibly by attenuating glial activation. However, further studies are needed to address this issue.

### KIT-13 enhanced neurogenesis in brain

**FIGURE 5:**
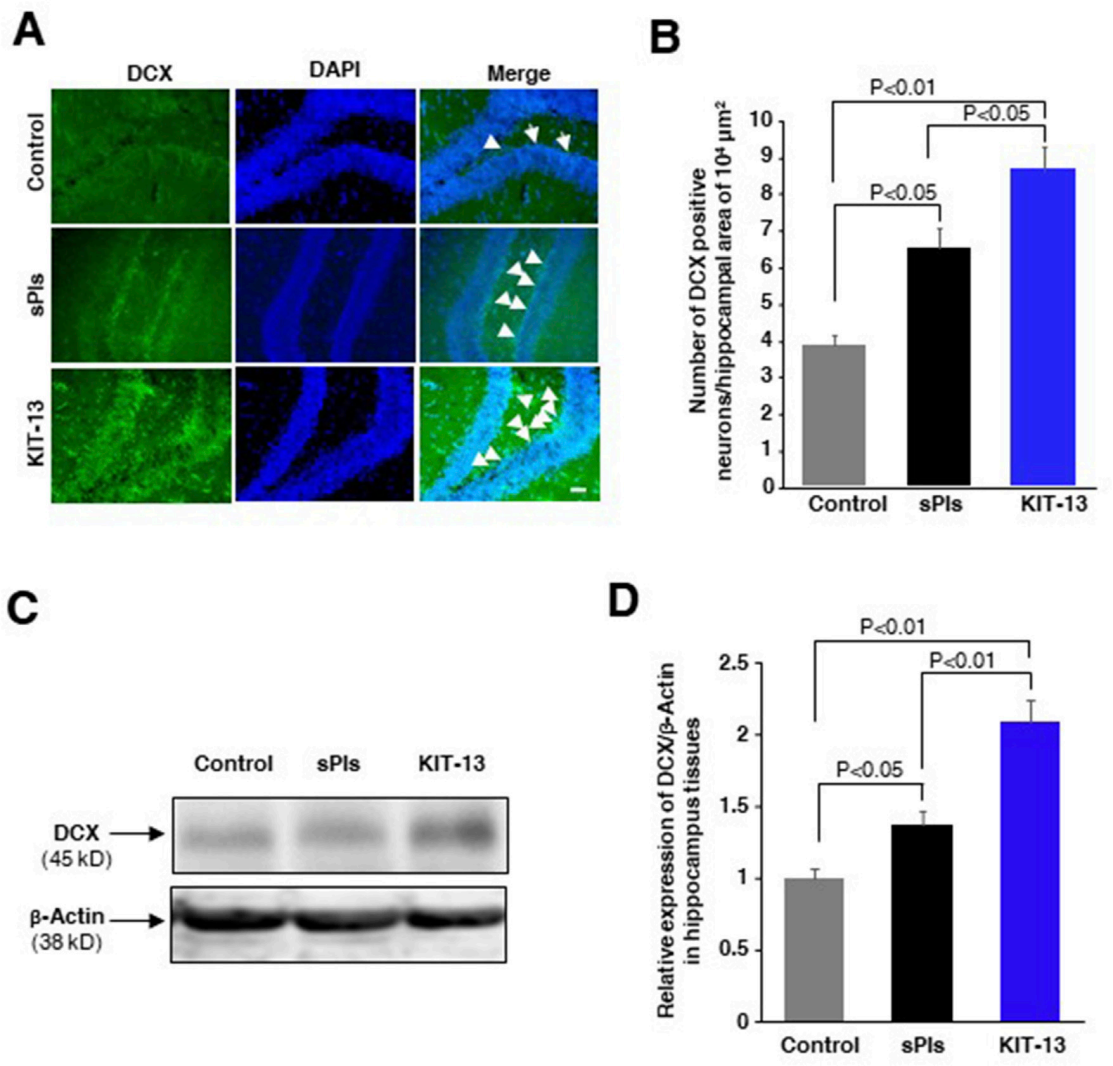
KIT-13 increased neurogenesis in adult mice hippocampus **(A)** Immunohistochemistry assays showing DCX-positive neurons in the hippocampal dentate gyrus area of adult male mice treated with KIT-13 and sPls (10 mg/kg body weight). After 24 h, brain slices were examined for DCX expression. DAPI was used to stain cell nuclei. Scale bar = 50 µm. **(B)** Quantification of DCX-positive neurons in the specified area of hippocampal tissues (n = 3). Values represent the mean ± SEM. *P*-values were calculated using ANOVA followed by post hoc Bonferroni tests. **(C)** Western blotting assays show the representative expression of DCX protein in the hippocampus tissues in the experiment of panel A. **(D)** The quantification data from Western blotting assays show the relative changes in the expression of DCX protein in hippocampus tissues. Beta-actin was used as a loading control for the proteins in each group. The data represent average values from three mice per group (n = 3), with error bars indicating the SEM. *P*-values were calculated using ANOVA followed by post hoc Bonferroni tests.

Oral administration of sPls showed enhanced neurogenesis associated with learning and memory in mice (Hossain et al., 2022b). To investigate whether KIT-13 treatments can similarly increase neurogenesis, we administered KIT-13 orally to adult male mice and performed immunohistochemistry assays to detect the neurogenesis marker protein doublecortin (DCX). Quantification of DCX-positive neurons in the hippocampal tissues revealed that KIT-13 treatment led to a substantial increase in neurogenesis compared to both the control and sPls groups (Figures 5A, B). The quantification data showed that the mean number of DCX-positive neurons in the KIT-13 group was significantly higher. In addition to the immunohistochemistry studies, Western blotting assays showed that KIT-13 treatments increased DCX protein expression in the hippocampus tissues (Figures 5C, D). These findings suggest that KIT-13 has a potent effect on promoting neurogenesis in the brain, surpassing the effects observed with sPls treatment alone. The enhanced neurogenesis observed with KIT-13 treatment may contribute to the improved learning and memory functions previously noted in behavioral tests. This highlights the potential of KIT-13 as a therapeutic agent for enhancing brain function through increased neurogenesis. The mechanism by which oral ingestion of KIT-13 and sPls enhances neurogenesis remains unknown in our studies. There could be direct effects if KIT-13 and sPls can enter the brain via the BBB, or indirect effects by modulating secondary signaling molecules in the peripheral system to activate the brain indirectly, including the activation of the vagus nerve system. Further studies are necessary to elucidate the detailed mechanism of action.

## Discussion

Our findings suggest that the new plasmalogens derivative, KIT-13, exhibits promising effects when compared to plasmalogens extracted from scallop (sPls). Its capacity to enhance cellular signaling, decrease cytokine expression, promote BDNF expression, inhibit neuronal apoptosis, improve spatial memory, alleviate neuroinflammation, reduce amyloid beta and enhance neurogenesis positions KIT-13 as a promising candidate for future research. It holds potential as a therapeutic alternative for various neurodegenerative diseases, including Alzheimer’s Disease. Given that oral ingestion of sPls showed promising benefits among patients with AD (Fujino et al., 2017; Fujino et al., 2020; Fujino et al., 2022), it is plausible that KIT-13 might exhibit even more substantial effects in this population.

While the direct application of scallop Plasmalogens and KIT-13 exhibited effects on cells, inducing cellular signaling and inhibiting the inflammatory effects of LPS, it remains uncertain whether the memory enhancement observed in this study is solely attributed to brain-related effects. Previous findings, where increased Pls content in the brain followed oral ingestion (Hossain et al., 2022b), suggest the potential for Pls to traverse the blood-brain barrier, exerting a direct influence. However, it is also plausible that some Pls may undergo degradation, leading to an increase in PUFA concentration, which could subsequently be utilized for Pls synthesis within the brain. Addressing these intricacies will necessitate further investigation in future studies. It is known that increased peripheral cytokines, often found in patients with AD, can exacerbate brain inflammation and impair memory (Kim et al., 2016; Su et al., 2016). As KIT-13 exhibited anti-inflammatory effects, it is plausible that a potential reduction in the peripheral cytokines following oral ingestion of KIT-13 could be one of the mechanisms underlying its memory improvement effects in mice and in human.

Given that KIT-13 is an alkyl-type Plasmalogen derivative in contrast to scallop Plasmalogens, which are alkenyl-type, the degradation of KIT-13 in the gut may be much lower. This difference could be one of the reasons for the superior effects of KIT-13 over scallop Plasmalogens. Additionally, scallop Plasmalogens are extracted from scallops and therefore consist of various types of Plasmalogens with different polyunsaturated fatty acids (PUFA) at the sn-2 position (Hossain et al., 2022b). However, KIT-13 only contains arachidonic acid in the sn-2 position, suggesting potential functional differences depending on the fatty acid composition of Plasmalogens. This could further be supported by the findings that different fatty acids in the sn-2 position had different effects in the activation of GPCR21, a receptor of Pls, in human NK cells (Hossain et al., 2022a).

KIT-13 is structurally different from the first identified plasmalogen derivative, PPI-1011. This PPI-1011 derivative features palmitic acid at the sn-1 position and DHA at the sn-2 position, showing promising results in reducing Parkinson’s-like symptoms in the MPTP-induced Parkinson’s model in animals (Gregoire et al., 2015; Bourque et al., 2018). KIT-13, to our knowledge, is the second reported alkyl-type Pls derivative and the first to demonstrate a memory improvement effect in mice. Besides alkyl Pls derivative, there is a vinyl-ether type Pls derivative, PPI-1040, which contains palmitic alcohol at the sn-1 position, docosahexaenoic acid (DHA) at the sn-2 position and a proprietary cyclic phosphoethanolamine group that increases the molecule’s stability at the sn-3 position (Fallatah et al., 2020). Future studies will be necessary to determine whether KIT-13 could have functional similarities to other Pls derivatives described early and whether any of these derivatives can cross the blood-brain barrier to explain a direct effect in the central nervous system. While most Pls derivatives were initially developed to increase Pls concentration to fight against pathological conditions in Pls deficiency models like RCDP, PD, and neuroinflammation, there is a strong likelihood that they could also provide benefits to adult individuals. This is due to the significant role in regulating cellular signaling, which is crucial for enhancing memory and other effects, including bolstering immunity against viruses (Braverman and Moser, 2012; Dorninger et al., 2020; Hossain et al., 2022a). This can further be supported by the recent clinical study showing that treatments with scallop-derived plasmalogens significantly improved mental concentration and alleviated negative mood states among young athletes aged between 18–22 years (Fujino et al., 2022).

The main drawback of these studies is the unclear mechanism of action regarding the effects of oral ingestion of KIT-13 on brain functions, such as memory improvements. It is still uncertain whether KIT-13 can cross the blood-brain barrier to increase Pls levels. Although KIT-13 is more stable than natural plasmalogens due to its alkyl nature, it is likely that KIT-13 could be broken down in the intestine to some extent, resulting in free fatty acids (such as arachidonic acid) and lyso-type plasmalogens. Therefore, it is possible that these degraded products may have biological effects. Additionally, KIT-13 might activate plasmalogen receptors (GPCRs) in peripheral tissues, leading to the modulation of peripheral substances that could affect the CNS. Thus, the oral ingestion of KIT-13 might impact the CNS indirectly, such as through activation of the vagus nerve system. Further studies are necessary to elucidate the mechanism of KIT-13 in improving memory and inhibiting neuroinflammation. However, our present studies provide valuable insights, indicating that this plasmalogen precursor exhibits better effects than scallop plasmalogens, suggesting that KIT-13 could be an attractive candidate for therapeutic use against neurodegenerative diseases.The promising results observed in this study pave the way for more comprehensive clinical trials and mechanistic studies to fully understand the therapeutic potential of KIT-13. Exploring the exact pathways and interactions of KIT-13 within the body will be crucial in developing it as a viable treatment option. Furthermore, the continued investigation into the comparative efficacy of different plasmalogen derivatives will enhance our ability to target specific neurodegenerative conditions more effectively. Ultimately, KIT-13 represents a significant advancement in plasmalogen research, with the potential to offer substantial benefits for patients suffering from Alzheimer’s disease and other related disorders.

## Data Availability

The raw data supporting the conclusions of this article will be made available by the authors, without undue reservation.
